# α-Mangostin suppresses lipopolysaccharide-induced invasion by inhibiting matrix metalloproteinase-2/9 and increasing E-cadherin expression through extracellular signal-regulated kinase signaling in pancreatic cancer cells

**DOI:** 10.3892/ol.2013.1290

**Published:** 2013-04-04

**Authors:** JIANGTAO YUAN, YAOLU WU, GUIFANG LU

**Affiliations:** 1 Department of General Surgery, The Affiliated Hospital of Yan’an University, Yan’an, Shaanxi 716000;; 2 Department of Gastroenterology, The First Affiliated Hospital of Medical College, Xi’an Jiao Tong University, Xi’an, Shaanxi 710061, P.R. China

**Keywords:** α-mangostin, pancreatic cancer, invasion, MMP-2, MMP-9, E-cadherin

## Abstract

Invasion and metastasis are major factors in the poor prognosis of pancreatic cancer, which remains one of the most aggressive and lethal diseases worldwide. α-mangostin, a major xanthone compound identified in the pericarp of mangosteen (*Garcinia mangostana*, Linn; GML), possesses unique biological activities, including antioxidant, antitumor and anti-inflammatory effects. Whether α-mangostin is able to inhibit the invasive ability of pancreatic cancer cells has not been elucidated. In the present study, α-mangostin was shown to inhibit the invasive ability of the pancreatic cancer cell lines MIAPaCa-2 and BxPC-3. The results showed that α-mangostin inhibited the growth of the pancreatic cancer cells in a dose- and time-dependent manner. At concentrations of <5 *μ*M, α-mangostin had no significant effects on cytotoxicity, but significantly inhibited the invasion and migration of pancreatic cancer cells and the expression of matrix metalloproteinase (MMP)-2 and MMP-9, while increasing the expression of E-cadherin. The present data also showed that α-mangostin exerted an inhibitory effect on the phosphorylation of extracellular-signal-regulated kinase (ERK). Furthermore, the reduction of ERK phosphorylation by small interfering RNA (siRNA) potentiated the effect of α-mangostin. Taken together, the data suggest that α-mangostin inhibited the invasion and metastasis of pancreatic cancer cells by reducing MMP-2 and MMP-9 expression, increasing E-cadherin expression and suppressing the ERK signaling pathway. The present study suggests that α-mangostin may be a promising agent against pancreatic cancer.

## Introduction

Pancreatic cancer is an aggressive malignant disease due to the lack of an early diagnosis and treatment options, and as such is the fourth leading cause of cancer mortality worldwide (1). Metastasis occurs frequently in the early stages of tumor development. In total, ∼75% of pancreatic cancer patients are diagnosed with the unresectable form of the disease (2). At present, gemcitabine, a standard first-line treatment for advanced pancreatic cancer, is extensively utilized, but offers only a modest benefit due to acquired chemoresistance and multiple adverse effects (3). In this context, the development of new effective therapeutic approaches for pancreatic cancer remains one of the most challenging goals in cancer research.

Numerous tropical plants have interesting biological activities with potential therapeutic applications. Traditional uses and later scientific findings have suggested that mangosteen (*Garcinia mangostana*, Linn; GML) is a potential candidate for an anticancer agent. GML belongs to the *Guttiferae* family and is termed ‘the queen of fruits’; it exerts a wide variety of pharmacological activities including antioxidant, antitumor and anti-inflammatory actions (4–6). Phytochemical studies have shown that the xanthones, as characteristic secondary metabolites of GML, are associated with these therapeutic roles (7). Of these, α-, β- and γ-mangostin are the most abundant representatives, as well as the most studied. The biological activities of α-mangostin, which have been demonstrated to include anti-proliferative and apoptotic effects on various cancer cells, including human leukemia and lung, colon and breast cancer (8–11), are the most significant. Although the anti-proliferative role of α-mangostin in malignant diseases has been increasingly recognized, its function in pancreatic cancer cells remains undetermined. The role of α-mangostin in inhibiting the invasion of pancreatic cancer has been particularly elusive.

Invasion and metastasis occurs through a complex, multistep process, which involves the invasion of cells from primary tumors into the circulation, migration of these cells to distant organs, adhesion to endothelial cells and infiltration into tissues. Matrix metalloproteinase (MMP)-2 and MMP-9 are presumed to be associated with the progression and invasion of various types of cancer cells and are highly expressed in pancreatic cancer. E-cadherin expression in pancreatic cancer is significantly lower than in normal pancreatic tissue and has been associated with lymph node and liver metastasis (12,13). It has been suggested that metastasis is responsible for the majority of failures in cancer treatment and is the major cause of mortality in patients with various cancer types. Therefore, in addition to minimizing the growth of existing tumors, treatments that limit the spread of cancer cells and block invasion have been pursued to enhance the survival of cancer patients. It was previously reported in various other cancer types that α-mangostin inhibited invasion by modulating MMP expression (9,14). The purpose of the present study was to demonstrate the effect of α-mangostin in inhibiting the invasion of pancreatic cancer cell lines. The molecular mechanisms of α-mangostin were also investigated in human pancreatic cancer cell invasion and metastasis.

## Materials and methods

### Reagents

α-mangostin (purity >98%) was acquired from ChromaDex Inc. (Irvine, CA, USA). Dimethylsulfoxide (DMSO) and 3-(4,5-dimethylthiazol-2-yl)-2,5-diphenyltetrazolium bromide (MTT) were purchased from Sigma Chemicals (St. Louis, MO, USA). Dulbecco’s modified Eagle’s medium (DMEM) and fetal bovine serum (FBS) were purchased from HyClone (Logan, UT, USA). Millicell culture plate inserts were purchased from Millipore (Bedford, MA, USA). Matrigel and the One-Step RT-PCR kit were purchased from BD Biosciences (Bedford, MA, USA). The antibodies against ERK, E-cadherin, MMP-9, MMP-2 and β-actin were purchased from Santa Cruz Biotechnology (Santa Cruz, CA, USA). All drug solutions were freshly prepared on the day of testing.

### Cell cultures and treatments

The human pancreatic cancer cell lines, BxPC-3 and MIAPaCa-2, were obtained from the American Type Culture Collection (Manassas, VA, USA). The study was approved by the Ethics Committee of Yan’an University, Yan’an, China. The cells were cultured in DMEM containing 10% dialyzed heat-inactivated FBS, 100 U/ml penicillin and 100 *μ*g/ml streptomycin in a humidified atmosphere of 5% CO_2_ at 37°C. In the invasion and migration assays, the cells were cultured in DMEM without FBS. A 100 mM solution of α-mangostin was prepared in DMSO and stored at −20°C. For the treatment, this solution was diluted in DMEM and added to the culture medium to the desired final concentration. Untreated cultures received an equivalent amount of the solvent (DMSO, 0.1%).

### Proliferation assay

Cell proliferation was determined by the MTT uptake method. BxPC-3 and MIAPaCa-2 cells were seeded onto 96-well plates at a density of 1×10^4^ cells per well and incubated overnight in 10% FBS medium. The cells were then incubated with various concentrations of α-mangostin in 0.1% DMSO. The cells that were treated with 0.1% DMSO alone were designated as the control group. Following incubation for 6, 12, 18, 24 and 48 h at 37°C, 15 *μ*l MTT solution (5 mg/ml in phosphate-buffered saline; PBS) was added to each well and the cells were incubated for 4 h at 37°C. A total of 100 *μ*l DMSO was then added to each well at 37°C. The optical density (OD) value at 490 nm was determined with a spectrophotometer (Bio-Rad, CA, USA). The results were calculated as the percentages relative to the controls. The proliferation inhibition rate = (1 − OD_sample_ / OD_control_) × 100.

### Cell migration assay

The cell migratory ability was evaluated with a wound-healing assay. Pancreatic cancer cells were seeded onto 24-well plates (1.0×10^5^ cells/500 *μ*l) and grown to 90% confluency. A wound line was then made between the confluent cells using sterile plastic pipette tips, and any cellular debris was removed by washing with PBS. The wounded monolayers were then incubated in the absence or presence of lipopolysaccharide (LPS; 5 *μ*g/ml) or α-mangostin (5 *μ*M) for 24 h and images were digitally captured. The migration area was measured using Image-Pro Plus 5.0 (Media Cybernetics Inc., Rockville, MD, USA).

### Cell invasion assay

The invasion of the pancreatic cancer cells was investigated using Transwell chambers. The Millicell culture plate filter inserts (8-*μ*m pore size, with a polyvinyl/pyrrolidone-free polycarbonate membrane) were coated with 100 *μ*l 1:10 diluted matrigel (5 mg/ml in cold DMEM). A thin continuous film was then formed on the top of the filter at 37°C for 4 h. The pancreatic cancer cells were detached from the culture flask and suspended in serum-free DMEM supplemented with 0.1% bovine serum albumin (BSA) and/or LPS/α-mangostin. The cells were seeded onto the upper compartments of the filter inserts. A total of 500 *μ*l DMEM with 10% FBS was placed into the lower compartments as a chemoattractant. Subsequent to 24 h of incubation, the filter inserts were removed from the plates. The cells on the surface of the filter in the upper compartment were removed by scraping with a cotton swab. The invasive cells on the lower surface of the filter were fixed in methanol and stained with the crystal violet reagent. The number of cells in five random fields was counted under a microscope (Olympus, Tokyo, Japan) (magnification, ×100). The data presented are the mean values obtained from three separate chambers.

### RT-PCR (reverse transcription polymerase chain reaction)

Total RNA from the cells was isolated using TRIzol reagent (Gibco-BRL. The first-strand cDNA was synthesized from 2 *μ*g total RNA using the RevertAid kit (Gibco BRL, Carlsbad, CA, USA). Quantitative normalization of the cDNA in each sample was performed using the expression of the β-actin gene as an internal control. The PCR primers that were used for the detection of MMP-2, MMP-9 and E-cadherin were synthesized as follows: MMP-2 (332 bp) forward, 5′-TGG TCC TGG TGC TCC TGG TG-3′ and reverse, 5′-GCT GCC TGT CGG TGA GAT TGG-3′; MMP-9 (111 bp) forward, 5′-TGG TCC TGG TGC TCC TGG TG-3′ and reverse, 5′-GCT GCC TGT CGG TGA GAT TGG-3′; E-cadherin (126 bp) forward, 5′-CAA TGG TGT CCA TGT GAA CA-3′ and reverse, 5′-CCT CCT ACC CTC CTG TTC G-3′; and β-actin (179 bp) forward, 5′-ATC GTG CGT GAC ATT AAG GAG AAG-3′ and reverse, 5′-AGG AAG AAG GCT GGA AGA GTG-3′. The PCR conditions were as follows: one cycle of denaturing at 94°C for 3 min, followed by 35 cycles at 94°C for 30 sec, 55°C for 30 sec and 72°C for 35 sec, follwed by a final extension at 72°C for 5 min. The PCR products were loaded onto 1.5% agarose gels and visualized with ethidium bromide under UV light.

### Western blotting

Briefly, 5×10^5^ cells were incubated on ice for 30 min in 0.5 ml ice-cold whole-cell lysate buffer. Any debris was removed by centrifugation. The protein content of the cells was determined and the cellular lysates were separated by 10% SDS-PAGE, then electro-transferred onto nitrocellulose membranes. Subsequent to being blocked with 5% skimmed milk in Tris-buffered saline with Tween 20 (TBST), the membranes were incubated with primary antibodies at 4°C overnight, followed by 1:2,000 horseradish peroxidase (HRP)-conjugated secondary antibodies for 2 h. Immunoreactive bands were visualized using an enhanced chemiluminescence kit (Amersham Pharmacia Biotech, Piscataway, NJ, USA). The western blotting signals were quantitated by densitometric analysis using TotalLab Nonlinear Dynamics Image analysis software (Nonlinear Dynamics, Durham, NC, USA).

### Small interfering RNA (siRNA) assay

siRNA oligos were used to inhibit the expression of ERK (ERK siRNA target sequence, 5′-UAAAGGUUAACAUCCGGUG-3′; Qiagen, Gaithersburg, MD, USA). The BxPC-3 cells (n=2×10^6^) were transfected with siRNA targeted against ERK (100 nm/l) or a control siRNA (Qiagen) using Lipofectamine 2000 (Invitrogen, Carlsbad, CA, USA). The cells were covered overnight prior to starvation. This was then followed by the treatment with α-mangostin (5 *μ*M) for 24 h. Finally, the cells were harvested for western blotting and an invasion assay.

### Statistical analysis

Each experiment was performed at least three times. Statistical analysis was performed using SPSS software (version 16.0; SPSS Inc. Chicago, IL, USA). The results are presented as the mean ± standard deviation (SD). The treatment differences were assessed using the Student’s t-test. Multiple group comparisons were performed with a one-way analysis of variance (ANOVA) followed by the Bonferroni post hoc test. Confirmation that the difference was statistically significant required the rejection of the null hypothesis, which would indicate that there was no difference between the mean value obtained from the replicate sets when P=0.05. Therefore, P<0.05 was considered to indicate a statistically significant difference.

## Results

### Effect of α-mangostin on the proliferation of pancreatic cancer cells

**Figure 1:**
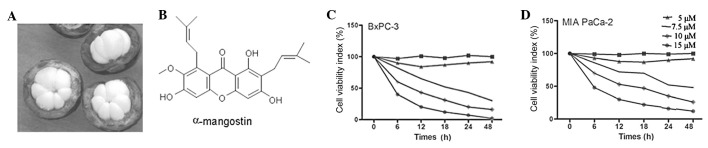
Effect of α-mangostin on the viability of BxPC-3 and MIAPaCa-2 cells. (A) *Garcinia mangostana* Linn (GML). (B) Chemical structure of α-mangostin. (C) The BxPC-3 and (D) MIAPaCa-2 cells were treated with various concentrations (0, 5, 7.5, 10 or 15 *μ*M) of α-mangostin for 6, 12, 18, 24 and 48 h. The number of surviving cells was directly proportional to the formazan level, which was measured spectrophotometrically at 563 nm. Values represent the mean ± SD of three independent experiments. ^*^P<0.05, compared with the untreated control.

The cytotoxicity of α-mangostin was first determined using the MTT assay. BxPC-3 and MIAPaCa-2 cells were treated with α-mangostin at various concentrations (0, 5, 7.5, 10 or 15 *μ*M) for 6, 12, 18, 24 or 48 h. The results demonstrated that the proliferative abilities of the BxPC-3 and MIAPaCa-2 cells were decreased by α-mangostin in a time- and dose-dependent manner. In addition, treatment with α-mangostin at ≤5 *μ*M exhibited no cytotoxic effects on the BxPC-3 or MIAPaCa-2 cells (Fig. 1). Therefore, this concentration of α-mangostin was used for the subsequent experiments.

### α-mangostin inhibits the migration of pancreatic cancer cells

**Figure 2:**
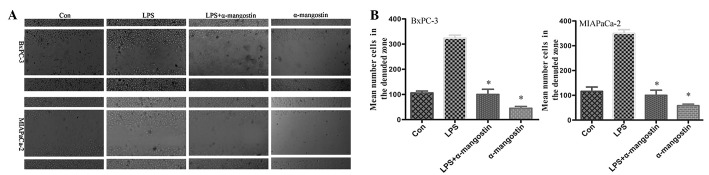
Effects of α-mangostin on the migration of BxPC-3 and MIAPaCa-2 pancreatic cancer cells. The cells were treated with LPS (5 *μ*g/ml) and/or α-mangostin (5 *μ*M) for 24 h, and were subjected to analyses for cell migration. (A) Images of migratory BxPC-3 and MIAPaCa-2 cells were captured under a microscope at ×40 magnification. Data are represented as the mean ± SD of three independent experiments. ^*^P<0.05 compared with the control. LPS, lipopolysaccharide; Con, control.

To evaluate the effect of α-mangostin on pancreatic cancer cell motility, the cell culture wound-healing assay, an established method for studying directional cell migration *in vitro,* was used. The serum-starved BxPC-3 and MIAPaCa-2 cells in the LPS (5 *μ*g/ml) group exhibited marked cell migration in the wound area 24 h after wounding, whereas the wounds treated with α-mangostin (5 *μ*M) showed delays in wound healing under the same conditions (Fig. 2). This result indicated that α-mangostin inhibited the migration of pancreatic cancer cells *in vitro*.

### α-mangostin inhibits the invasion of pancreatic cancer cells

**Figure 3:**
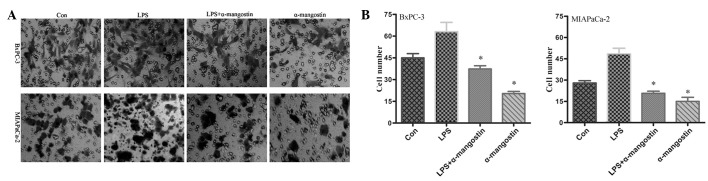
Effects of α-mangostin on the invasion of BxPC-3 and MIAPaCa-2 pancreatic cancer cells. The cells were treated with LPS (5 g/ml) and/or α-mangostin (5 M) for 24 h, then subjected to analyses for cell invasion. LPS significantly (P<0.05) stimulated cell invasion, an effect which was completely blocked by α-mangostin. (A) Images showing the bottom side of the filter inserts with cells that have migrated through the filter pores. (B) Columns in the graph represent the count analysis. Images of the invasive BxPC-3 and MIAPaCa-2 cells were captured under a microscope at ×100 magnification. Data are represented as the mean ± SD of three independent experiments. ^*^P<0.05 compared with the LPS group. LPS, lipopolysaccharide; Con, control.

To examine the potential effects on cell invasiveness, an invasion assay was performed on the BxPC-3 and MIAPaCa-2 cells. The motile phenotypes of the cells treated with LPS and the combination of LPS plus α-mangostin were evaluated. Following treatment with LPS alone, the number of invasive cells increased significantly compared with the untreated cells. The number of invasive cells was significantly reduced in the cells co-treated with LPS and α-mangostin (P<0.05) (Fig. 3). These results suggested that α-mangostin blocked the effect of LPS, which increased the invasiveness of the pancreatic cancer cells. This observation revealed that α-mangostin may be an effective inhibitor of the invasion of pancreatic cancer cells.

### Effects of α-mangostin on the mRNA and protein expression of MMP-2, MMP-9 and E-cadherin

**Figure 4:**
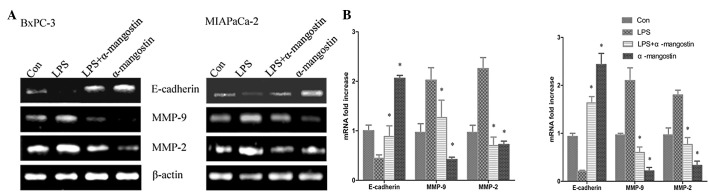
α-mangostin prevented the LPS-induced decrease in the expression of E-cadherin mRNA and the increase in the expression of MMP-9 and MMP-2 mRNA. (A) The mRNA expression levels of E-cadherin, MMP-9 and MMP-2 in BxPC-3 and MIAPaCa-2 cells were determined by RT-PCR. (B) Quantification of the mRNA levels. Data from at least three independent experiments with duplicate determinations are expressed as the mean ± SEM. ^*^P<0.05 was considered to indicate statistically significant differences for LPS + α-mangostin group compared with the LPS group. LPS, lipopolysaccharide; MMP, matrix metalloproteinase; Con, control.

**Figure 5:**
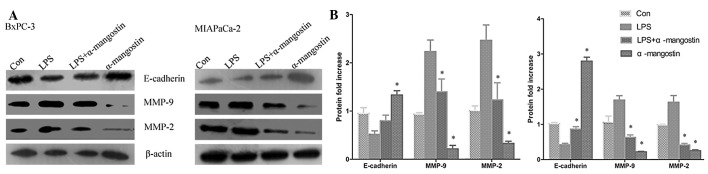
α-mangostin prevented the LPS-induced decrease in the expression of E-cadherin protein and LPS-induced increase in the expression of MMP-9 and MMP-2 protein. (A) Protein expression levels of E-cadherin, MMP-9, and MMP-2 in BxPC-3 and MIAPaCa-2 cells were determined via western blotting. (B) Quantification of the protein levels. Data from at least three independent experiments with duplicate determinations are expressed as the mean ± SEM. ^*^P<0.05 was considered to indicate considered statistically significant differences for LPS + α mangostin group compared with the LPS group. LPS, lipopolysaccharide; MMP, matrix metalloproteinase; Con, control.

A number of studies have investigated the importance of E-cadherin in interations between the cells and the ECM, which may inhibit cell migration and invasion. Metastasis has been shown to be accompanied by various physiological alterations involved in the degradation of the ECM, including the overexpression of proteolytic enzyme activity. To further confirm the inhibitory effects of α-mangostin on LPS-induced migration and invasion, the mRNA and protein expression levels of MMP-9, MMP-2 and E-cadherin were sequentially analyzed using semi-quantitative RT-PCR analysis and western blotting, respectively. The semi-quantitative PCR results (Fig. 4) indicated that the mRNA levels of MMP-9 and MMP-2 were significantly increased, while E-cadherin was significantly suppressed by LPS treatment (P<0.05). The western blotting (Fig. 5) showed that the expression of the E-cadherin protein was significantly downregulated in the LPS group compared with the control (P<0.05), whereas MMP-9 and MMP-2 protein expression was significantly increased (P<0.05). Notably, α-mangostin reversed this phenomenon as induced by LPS, causing the re-induction of E-cadherin and the inhibition of MMP-9 and MMP-2 expression (P<0.05) (Figs. 4 and 5). These results further suggested that α-mangostin has an inhibitory effect on cellular migration and invasion.

### ERK signaling has a key role in the effect of α-mangostin on the expression of MMP-2, MMP-9 and E-cadherin

**Figure 6:**
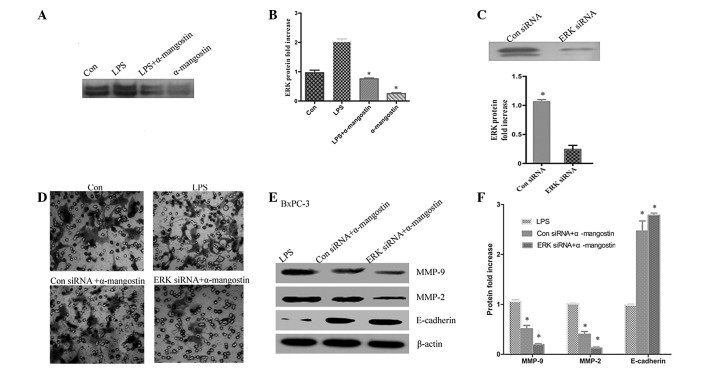
ERK signaling is key to the effect of α-mangostin on the expression of MMP-2, MMP-9 and E-cadherin. (A) α-mangostin reversed LPS-induced ERK1/2 activation. (B) Quantification of protein levels. (C) The efficacy of ERK siRNA for knocking down ERK protein was demonstrated by western blotting. (D) Cells were treated with LPS (5 *μ*g/ml), α-mangostin (5 *μ*M) and/or ERK siRNA for 24 h, then subjected to analyses for cell invasion. (E) BxPC-3 cells were treated with LPS (5 *μ*g/ml) and/or α-mangostin (5 *μ*M), with or without ERK siRNA. After 24 h, the E-cadherin, MMP-9 and MMP-2 protein expression levels were detected using western blotting. (F) Quantification of protein levels. Data from at least three independent experiments with duplicate determinations are expressed as the mean ± SEM. ^*^P<0.05 was considered to indicate statistically significant results for LPS + α-mangostin group compared with the LPS group LPS, lipopolysaccharide; MMP, matrix metalloproteinase; siRNA, small interfering RNA; Con, control.

Previous studies have reported that α-mangostin is able to inhibit cancer cell invasion by suppressing the ERK1/2 signaling pathway (9). To further investigate whether the inhibition of LPS-induced MMP-2 and MMP-9 expression by α-mangostin occurred via ERK1/2 activation, ERK1/2 phosphorylation was detected by western blotting. The results showed that the phosphorylation of ERK1/2 was significantly upregulated in the LPS group compared with the control (P<0.05), whereas α-mangostin reversed the LPS-induced ERK1/2 activation (Fig. 6A and B). To further demonstrate that α-mangostin inhibited invasion through the ERK signaling pathway, the BxPC-3 cells were transiently transfected with ERK siRNA. The efficacy of the ERK siRNA for knocking down ERK was demonstrated by western blotting. It was observed that the ERK protein level (Fig. 6B) was barely detectable in the ERK siRNA-transfected cells compared with the control siRNA-transfected cells (Fig. 6C). Subsequently, the effects of α-mangostin were determined by comparing results in the presence or absence of α-mangostin. The protein expression levels of MMP-2, MMP-9 and E-cadherin were evaluated by western blotting. As shown in Fig. 6E and F, sole treatment with α-mangostin reduced the LPS-induced expression levels of MMP-2 and MMP-9 and increased the expression of E-cadherin. The combined treatment (ERK siRNA and α-mangostin) was able to markedly reduce the expression levels of MMP-2 and MMP-9 compared with LPS treatment alone (P<0.05). Additionally, invasion and migration was reduced in the ERK siRNA group compared with the LPS group (Fig. 6D). These data indicate that the ERK signaling pathway may have a key role in the inhibition of LPS-induced invasion and migration by α-mangostin in pancreatic cancer cells.

## Discussion

Cancer metastasis refers to the spread of cancer cells from the primary neoplasm to distant sites and the growth of secondary tumors at sites that are distant from the primary tumor. Pancreatic cancer is a highly metastatic tumor. Blocking its invasion may lead to new, effective treatments for pancreatic cancer. Active compounds with anti-invasive and anti-metastatic properties have defined a new catalogue of chemotherapeutic agents, including α-mangostin, which have important roles in cancer treatment.

In previous studies, α-mangostin was able to inhibit the invasion of lung and breast cancer (9,14); the invasion inhibiting effect was greater in highly invasive carcinoma cells, but independent of its anti-proliferative action. The present study has shown that BxPC-3 and MIAPaCa-2 cell invasion and migration may be induced by LPS. Treatment with α-mangostin was able to effectively inhibit the LPS-induced effect, similar to the results of previous studies. These pancreatic cancer cells showed reduced migratory ability and delays in wound healing following incubation with α-mangostin. In the invasion assay, the average number of cells invading the lower chamber in 24 h was less than the control group following incubation with α-mangostin. α-mangostin not only restored metastatic activation, but also blocked the expression of MMP-9 and MMP-2 and upregulated E-cadherin. It was also demonstrated that ERK signaling is required for α-mangostin-mediated inhibition of the metastatic activation of the BxPC-3 and MIAPaCa-2 cells. These findings aid in the understanding of the mechanism by which α-mangostin acts to inhibit cancer cell invasiveness.

Invasion and metastasis is a highly complicated process that requires the interaction of numerous cell types, including connective tissue and blood vessel components within various organs (15,16). The invasion of tumor cells into tumor-associated stroma and the subsequent metastasis are central events in tumor progression. The metastasis of cancer cells involves changes in cell adhesion, rearrangement of the ECM, anoikis-suppression and reorganization of the cytoskeleton (17). Multiple molecular mechanisms are involved in this process. E-cadherin is a key molecule involved in regulating adhesion between cells through the binding of E-cadherin in the cytoplasm to catenin, which integrates the cytoskeleton of adjacent cells. The loss of E-cadherin provides cancer cells with an invasive ability (18). The association between MMP expression and the invasive activity of various types of cancer has been well documented. MMP-2 and MMP-9 enzymes are capable of degrading type IV collagen, which is a major constituent of the basement membrane (19,20).

α-mangostin has been demonstrated to be an inhibitory agent for MMP-2 and MMP-9 in lung and breast cancer (9,14). In the present study, it was observed that MMP-9 and MMP-2 mRNA expression in two pancreatic cancer cell lines decreased following treatment with α-mangostin, indicating that the inhibition of pancreatic cancer cell invasion by α-mangostin may be partly mediated by the downregulation of MMP-9 and MMP-2 expression. In addition, the effects of α-mangostin treatment on E-cadherin expression have not been reported previously. In the present study, E-cadherin was observed to be expressed at low levels in the two pancreatic cancer cell lines, but following incubation with α-mangostin, the expression of E-cadherin was significantly increased. This may be another mechanism by which α-mangostin inhibits the invasion of pancreatic cancer cells. Han *et al*(21) reported that genistein decreased the invasion of MIAPaCa-2 cells, upregulated the mRNA and protein expression of E-cadherin and reversed the characteristic morphology of epithelial-mesenchymal trans-differentiation (EMT). Thus, α-mangostin may reverse EMT by increasing E-cadherin expression. This is likely to require elucidation in future investigations.

Previously, α-mangostin has been shown to suppress the phosphorylation of ERK, leading to the inhibition of MMP-2 and MMP-9 in pancreatic cancer cells (10). In the present study, α-mangostin was also observed to directly inhibit ERK activity. Moreover, the combined treatment using ERK siRNA and α-mangostin was able to markedly reduce the expression of MMP-2 and MMP-9, while increasing that of E-cadherin. This result suggests that α-mangostin may target proteins upstream of ERK.

## References

[1] Bosetti C, Bertuccio P, Negri E, La Vecchia C, Zeegers MP, Boffetta P (2012). Pancreatic cancer: overview of descriptive epidemiology. Mol Carcinog.

[2] Książkiewicz M, Markiewicz A, Zaczek AJ (2012). Epithelial-mesenchymal transition: a hallmark in metastasis formation linking circulating tumor cells and cancer stem cells. Pathobiology.

[3] Brunner T (2012). Gemcitabine in the chemoradiotherapy for locally advanced pancreatic cancer: a meta-analysis. Strahlenther Onkol.

[4] Ngawhirunpat T, Opanasopi P, Sukma M (2010). Antioxidant, free radical-scavenging activity and cytotoxicity of different solvent extracts and their phenolic constituents from the fruit hull of mangosteen (*Garcinia mangostana*). Pharm Biol.

[5] Martínez-Abundis E, García N, Correa F, Hernández-Reséndiz S, Pedraza-Chaverri J, Zazueta C (2010). Effects of alpha-mangostin on mitochondrial energetic metabolism. Mitochondrion.

[6] Bumrungpert A, Kalpravidh RW, Chuang CC (2010). Xanthones from mangosteen inhibit inflammation in human macrophages and in human adipocytes exposed to macrophage-conditioned media. J Nutr.

[7] Sánchez-Pérez Y, Morales-Bárcenas R, García-Cuellar CM (2010). The alpha-mangostin prevention on cisplatin-induced apoptotic death in LLC-PK1 cells is associated to an inhibition of ROS production and p53 induction. Chem Biol Interact.

[8] Lee YB, Ko KC, Shi MD (2010). Alpha-Mangostin, a novel dietary xanthone, suppresses TPA-Mediated MMP-2 and MMP-9 expressions through the ERK signaling pathway in MCF-7 human breast adenocarcinoma cells. J Food Sci.

[9] Hung SH, Shen KH, Wu CH, Liu CL, Shih YW (2009). Alpha-Mangostin suppresses PC-3 human prostate carcinoma cell metastasis by inhibiting matrix metalloproteinase-2/9 and urokinase-plasminogen expression through the JNK signaling pathway. J Agric Food Chem.

[10] Matsumoto K, Akao Y, Ohguchi K (2005). Xanthones induce cell-cycle arrest and apoptosis in human colon cancer DLD-1 cells. Bioorg Med Chem.

[11] Lu X, Kang Y (2010). Hypoxia and hypoxia-inducible factors: master regulators of metastasis. Clin Cancer Res.

[12] Higgins DF, Kimura K, Bernhardt WM (2007). Hypoxia promotes fibrogenesis in vivo via HIF-1 stimulation of epithelial-to-mesenchymal transition. J Clin Invest.

[13] Shih YW, Chien ST, Chen PS, Lee JH, Wu SH, Yin LT (2010). Alpha-mangostin suppresses phorbol 12-myristate 13-acetate-induced MMP-2/MMP-9 expressions via alphavbeta3 integrin/FAK/ERK and NF-kappaB signaling pathway in human lung adenocarcinoma A549 cells. Cell Biochem Biophys.

[14] Patel P, Chen EI (2012). Cancer stem cells, tumor dormancy, and metastasis. Front Endocrinol (Lausanne).

[15] Nakayama K, Nakayama N, Katagiri H, Miyazaki K (2012). Mechanisms of ovarian cancer metastasis: biochemical pathways. Int J Mol Sci.

[16] Wilmanns C, Steinhauer S, Großmann J, Schmitt-Gräff A, Ruf G (2012). Cooperate concept of metastasis: site-specific requirement of activated differentiation and dynamic deterioration. Cancer Metastasis Rev.

[17] Buda A, Pignatelli M (2011). E-cadherin and the cytoskeletal network in colorectal cancer development and metastasis. Cell Commun Adhes.

[18] Kowluru RA, Zhong Q, Santos JM (2012). Matrix metalloproteinases in diabetic retinopathy: potential role of MMP-9. Expert Opin Investig Drugs.

[19] Romi F, Helgeland G, Gilhus NE (2012). Serum levels of matrix metalloproteinases: implications in clinical neurology. Eur Neurol.

[20] Han L, Zhang HW, Zhou WP, Chen GM, Guo KJ (2012). The effects of genistein on transforming growth factor-β1-induced invasion and metastasis in human pancreatic cancer cell line Panc-1 in vitro. Chin Med J (Engl).

